# Myocardial mapping of T1 and T2 with 3D-QALAS - precision of independent and dependent scans in healthy subjects

**DOI:** 10.1186/1532-429X-18-S1-P11

**Published:** 2016-01-27

**Authors:** Sofia Kvernby, Marcel Warntjes, Jan E Engvall, Carl Johan Carlhall, Tino Ebbers

**Affiliations:** 1 Institution for Medicine and Health Science, Linköping, Sweden; 2 Center for Medical Image Science and Visualization (CMIV), Linköping, Sweden

## Background

Recently, a new method for simultaneous myocardial T1- and T2 relaxation times mapping of the whole left ventricular myocardium in a single breath hold, 3D-QALAS, has been proposed and verified in-vitro [1]. The clinical utility of quantitative methods is dependent on a good precision to allow differentiation between healthy and pathological myocardium. The aim of this study was to investigate the in-vivo precision of 3D-QALAS in healthy volunteers.

## Methods

Ten healthy subjects underwent four scan blocks during the same day on a Philips Ingenia 3T system. Each subject was removed from the bore and repositioned between the first and the second scan block to achieve independent measurements and thus investigate repeatability. The scan protocol for the first, second and third scan blocks consisted of three MOLLI acquisitions (apical, mid-ventricular and basal), three T2-GraSE acquisitions (apical, mid-ventricular and basal) and one 3D-QALAS acquisition. The scan protocol for the fourth scan block consisted of eight mid-ventricular MOLLI acquisitions, eight mid-ventricular GraSE acquisitions and eight 3D-QALAS acquisitions, with the aim to investigate precision by using standard deviations of repeated measurements.

T1- and T2-maps from 3D-QALAS were generated using SyMRI (SyntheticMR, Sweden) and maps from the reference methods were generated directly on the scanner console. The generated T1- and T2-maps from all methods were analyzed using Segment v1.9 R3644.

## Results

**Figure 1:**
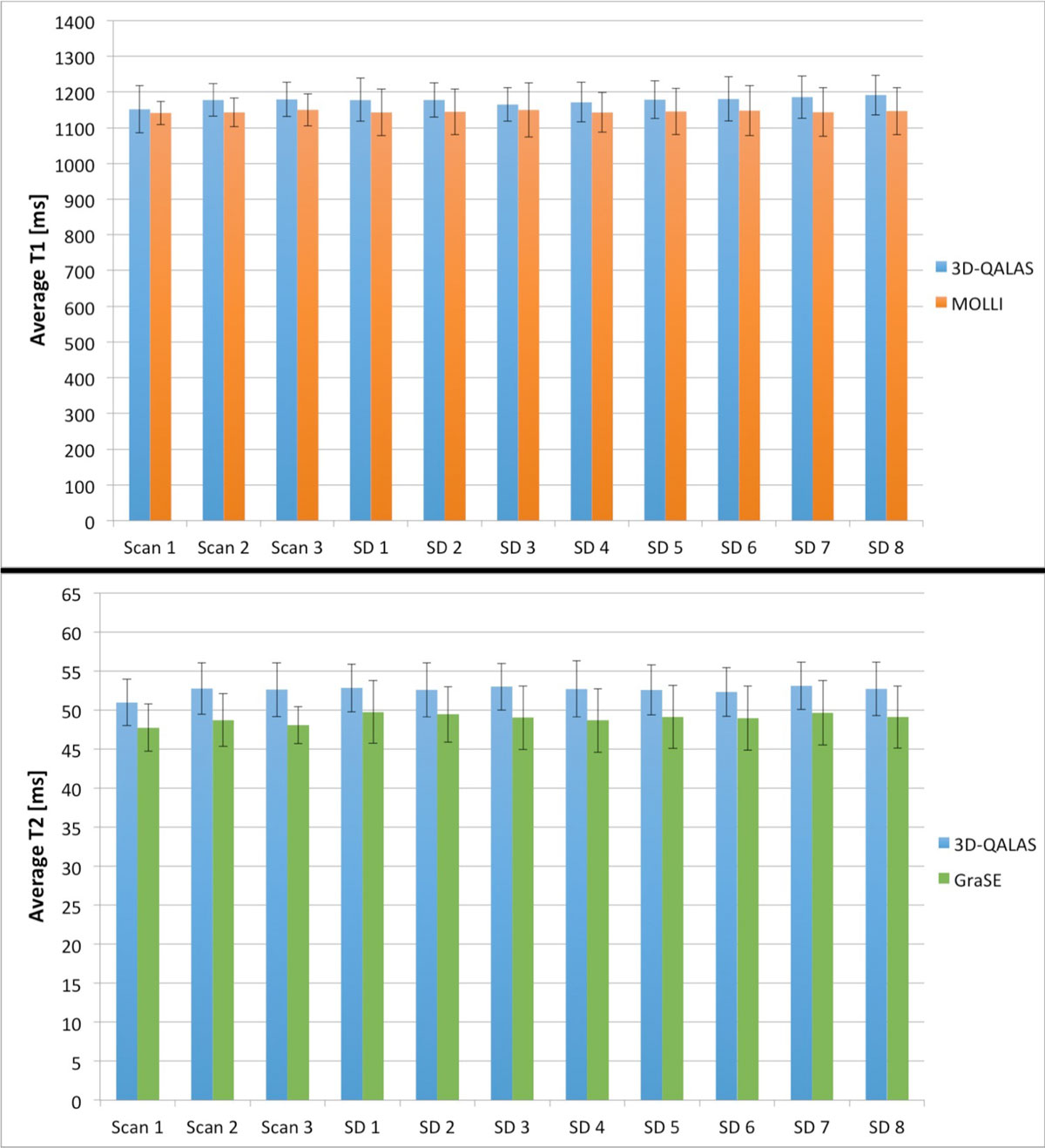
**Average myocardial relaxation times values (upper: T1, lower: T2) and standard deviations from the group of ten healthy volunteers for each scan**.

Myocardial relaxation times from the two independent scans, first versus the second scan block, showed a less good correlation than myocardial relaxation times from the second versus the third scan block, table 1, for all methods. Average values and SD for the group of subjects for each scan are shown in figure 1.

**Table 1:** Comparative result from the first three scans, based on ten healthy volunteers. Mean difference is expressed as the average difference from all healthy volunteers.

	Scan 1 vs. scan 2 Independent scans	Scan 2 vs. scan 3 Dependent scans
3D-QALAS (T1) Pearson correlation Mean difference, 95% CI [ms]	. r = 0.782 Δ = -26.1, (-56.1; 3.8)	. r = 0.915 Δ = -1.7, (-14.0; 10.5)
MOLLI Pearson correlation Mean difference, 95% CI [ms]	. r = 0.583 Δ = -1.6, (-22.7; 19.5)	. r = 0.812 Δ=-7.0, (-23.3; 9.3)
3D-QALAS (T2) Pearson correlation Mean difference, 95% CI [ms]	. r = 0.624 Δ = -1.8, (-3.0; -0.6)	. r = 0.648 Δ = 0.1, (-1.3; 1.6)
GraSE Pearson correlation Mean difference, 95% CI [ms]	. r = 0.588 Δ = -1.0, (-2.4; 0.4)	. r = 0.903 Δ = 0.7, (-0.2; 1.5)

Average myocardial relaxation time values and SD from eight repeated acquisitions within the group of subjects were 1178 ± 18.5 ms (1.6%) for T1 with 3D-QALAS, 52.7 ± 1.2 ms (2.3%) for T2 with 3D-QALAS, 1145 ± 10.0 ms (0.9%) for T1 with MOLLI and 49.2 ± 0.8 ms (1.6%) for T2 with GraSE. Intraclass correlation analysis for the consecutive scans showed that both 3D-QALAS and the reference methods have a very high reliability.

## Conclusions

Precision has been investigated between two independent scans, between two dependent scans and as standard deviation of eight consecutive scans in ten healthy volunteers. All methods (MOLLI, GraSE and 3D-QALAS) showed good precision. The standard deviation of eight consecutive scans was slightly better using MOLLI for T1 and GraSE for T2 than for 3D-QALAS, while the correlation for two independent scans was slightly better using 3D-QALAS.
